# Hypermethylation of the HIC1 promoter and aberrant expression of HIC1/SIRT1 contribute to the development of thyroid papillary carcinoma

**DOI:** 10.18632/oncotarget.12936

**Published:** 2016-10-26

**Authors:** Wenyi Wu, Liting Zhang, Jianqing Lin, Hanwei Huang, Bai Shi, Xingong Lin, Zhongxin Huang, Chaoyang Wang, Jianlong Qiu, Xiaolong Wei

**Affiliations:** ^1^ Department of general surgery, The Second Affiliated Hospital of Fujian Medical University, Quanzhou, Fujian, China; ^2^ Endocrine Department, The 180th Military Hospital of Chinese Peoples, Liberation Army, Quanzhou, Fujian, China; ^3^ Endocrine Department, Affiliated Zhongshan Hospital of Guangdong Medical College, Zhongshan, Guangdong, China; ^4^ Department of Pathology, The Second Affiliated Hospital of Fujian Medical University, Quanzhou, Fujian, China; ^5^ Department of Pathology, Cancer Hospital of Shantou University Medical College, Shantou, Guangdong, China

**Keywords:** HIC1, SIRT1, papillary thyroid carcinoma, promoter hypermethylation

## Abstract

Hypermethylation leading to the loss of hypermethylated in cancer-1 *(HIC1)* gene expression occurs in many different types of human cancer. HIC1 is a transcriptional repressor that directly binds to the promoter region of NAD-dependent deacetylase sirtuin-1 (SIRT1). SIRT1 functions in cell growth, is anti-apoptotic, protect neurons, functions in senescence, and regulates energy restriction. Epigenetic modification and dysregulation affecting the HIC1/SIRT1 axis is potentially important for the development of malignancies. However, the importance of HIC1 expression in the development of papillary thyroid carcinoma, especially in Chinese patients, is uncertain. Therefore, we assessed the level of methylation in the *HIC1* promoter and the mRNA and protein expression levels of HIC1 and SIRT1 in human thyroid papillary carcinoma and tumor adjacent control tissues. The demethylation reagent 5-aza-2′-deoxyctidine (5-aza-dc) and an HIC1 overexpression plasmid were used to manipulate the HIC1/SIRT1 pathway, and the effects on cell senescence, apoptosis, and cell cycle progression were assessed. Compared to normal thyroid tissue, thyroid tumors had lower expression of HIC1 and higher SIRT1 expression. The level of *HIC1* methylation was also higher in thyroid carcinoma tissues than adjacent tissues. HIC1 expression was closely correlated with patient age and tumor progression. Restoration of HIC1 expression through an overexpression plasmid or 5-aza-dC treatment reduced SIRT1 expression and cell proliferation, and led to senescence, cell cycle arrest, and apoptosis. Aberrant expression of HIC1/SIRT1 and hypermethylation of the *HIC1* promoter may be critical for the development and progression of papillary thyroid cancer.

## INTRODUCTION

Thyroid cancer originating from the thyroid epithelial cells is the most common endocrine malignancy. The incidence of thyroid cancer in the general population has gradually increased, and papillary thyroid carcinoma, which accounts for 80–85% of thyroid malignancies, is the most common form of thyroid cancer [1, 2]. While the etiology of thyroid cancers remains somewhat unclear, it is likely related to the interplay of environmental and genetic factors such as tumor suppressor gene promoter methylation.

Epigenetic gene silencing can disrupt the function of tumor suppressor genes, thereby promoting the activation of oncogenic genetic mutations [3]. Hypermethylated in cancer-1 (*HIC1*), a tumor suppressor gene essential to mammalian development [4], is located in the chromosomal regions 17p13.3, and is telomeric to the TP53. In many different human cancers (including prostate [5–7], hepatocellular [8, 9], pancreatic [10], renal cell carcinoma [11], breast cancers [12] and esophageal cancers [13], *HIC1* is epigenetically inactivated but not mutated [14]. The methylation status of the *HIC1* promoter is also associated with tumorigenesis and poor survival in patients with medulloblastomas [15]. In a pancreatic cancer model, restoration of HIC1 function can be accomplished by forced suppression by demethylation of the promoter and prevents cancer cell formation and reduces the aggressiveness of the tumors [10]. HIC1 binds to the deacetylase that regulates expression of NAD-dependent deacetylase sirtuin-1 (SIRT1), forming a transcriptional repression complex, which subsequently binds to the SIRT1 promoter and suppresses its transcription [16, 17]. SIRT-1 is a protomember of the sirtuin family [18] and is known to function in cell growth, be anti-apoptotic, protect neurons, function in senescence, and regulate energy restriction [19, 20]. Inactivation of *HIC1* upregulates SIRT1 expression in both cancerous and normal cells and promotes tumorigenesis [16, 21]. Recent studies indicate that *HIC1* methylation causes abnormal overexpression of SIRT, which contributes to the development and progression of breast [16], lung [17], and pancreatic cancers [10].

In papillary thyroid carcinoma, the methylation and expression patterns of *HIC1* have not yet been described and it is unclear whether the HIC1/SIRT1 pathway is involved in the development and progression of disease. Therefore, we investigated the methylation patterns of the *HIC1* promoter in primary samples from thyroid papillary carcinoma tumors and tissue adjacent to the tumors. We hypothesized that *HIC1* promoter methylation would cause abnormal expression of HIC1/SIRT1, thereby enhancing the development and progression of papillary thyroid carcinoma.

## RESULTS

### Expression of HIC1 and SIRT1 in thyroid carcinomas and normal thyroid tissues

**Figure 1:**
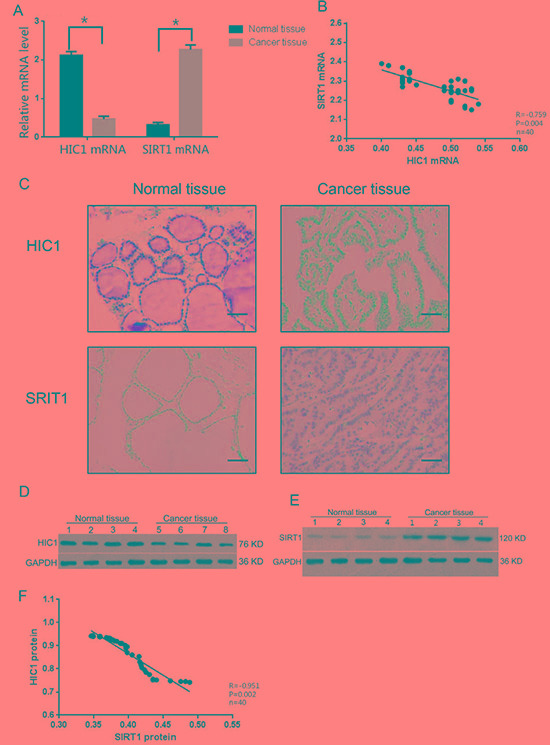
Expression HIC1 and SIRT1 in papillary thyroid cancer tissue The mRNA and protein levels of HIC1 and SIRT1 were assessed in papillary thyroid cancer tissues and adjacent histologically normal tissue (*n* = 40 each) by qRT-PCR (**A**–**B**), immunohistochemistry, (Original magnification, 200×) Scale bar = 50 μm (**C)**, and Western blot (**D**–**F**). Western blot was used to detect HIC1, SIRT1, and GAPHD expression. *p < 0.05 compared with noncancerous tissue with the Student's *t-test*. Pearson correlation analysis was used for panels B and F.

The mRNA expression levels of HIC1 and SIRT1 were measured in samples from thyroid carcinomas and adjacent normal thyroid tissues. The relative HIC1 mRNA expression was significantly lower in papillary thyroid carcinomas (0.47 ± 0.07) than in the paired normal thyroid tissues (2.12 ± 0.10). Similarly, the relative SIRT1 mRNA expression in papillary thyroid carcinomas (2.27 ± 0.12) was significantly higher than in the paired normal thyroid tissues (0.32 ± 0.06) (Figure 1A). A negative correlation between the mRNA expression levels of HIC1 and SIRT1 was observed in the papillary thyroid carcinomas (Figure 1B). The protein expression levels of HIC1 and SIRT1 were measured by immunohistochemistry. In normal thyroid tissues, HIC1 was expressed in both the nuclei and the cytoplasm of normal thyroid tissues, although nuclear expression was dominant. In the papillary thyroid carcinoma tissues, HIC1 expression was relatively low compared to the paired normal thyroid tissue and only expressed in the nucleus (Figure 1C). Immunohistochemical analysis of SIRT1 expression confirmed low levels of nuclear protein in normal thyroid tissues, and staining in both the nucleus (dominant) and cytoplasm in papillary thyroid carcinomas. The expression level of nuclear SIRT1 in papillary thyroid carcinomas was significantly greater than the adjacent normal thyroid tissues (Figure 1C). Statistical analysis confirmed that HIC1 expression in primary papillary thyroid carcinomas was significantly correlated with lymph node metastasis of thyroid cancer, age, and TNM staging, but not with sex, tumor size, or tumor capsular invasion (Table 1).

**Table 1:** Patient characteristics stratified by HIC1 expression in human papillary thyroid cancer tissues

Clinical feature	*n*	HIC1		*P*
		−	+	
Lymph node metastasis				
Yes	18	14	4	0.038
No	22	10	12	
Sex				
Male	14	8	6	0.787
Female	26	16	10	
Age (year)				
< 45	25	12	13	0.046
≥ 45	15	12	3	
Tumor size (cm)				
< 3	24	14	10	0.792
≥ 3	16	10	6	
TNM stage				
I–II	28	12	16	0.018
III–V	12	10	2	
Capsule invasion				
Yes	17	9	8	0.433
No	23	15	8	

Significant differences were observed in the levels of HIC1 and SIRT1 protein expression by Western blot analysis in the thyroid cancer tissues and adjacent normal tissues. HIC1 protein expression in the papillary thyroid carcinomas (0.42 ± 0.07) was lower than in the adjacent normal tissues (0.94 ± 0.11; *p <* 0.01) and the inverse was true for SIRT1 protein expression (Figure 1D–1E). Consistent with the mRNA results, there was also a negative correlation between HIC1 and SIRT1 protein expression in the papillary thyroid carcinomas (Figures 1F, *p <* 0.05).

### Hypermethylation of the HIC1 gene in thyroid cancer

**Figure 2:**
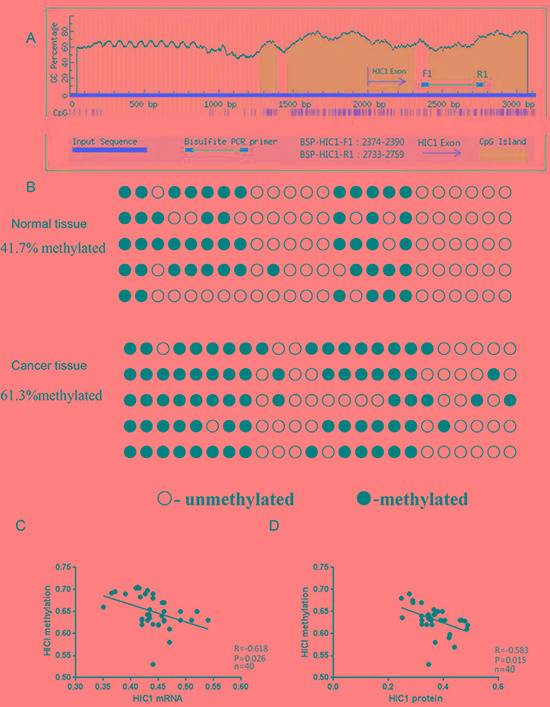
Analysis of HIC1 promoter methylation, mRNA expression, and protein expression in papillary thyroid cancer tissue (**A**) The CpG islands and locations of the BGS primers in the promoter of *HIC1* are shown. BSP-HIC1-F1: BGS forward primer, BSP-HIC1-R1: BGS reverse primer. (**B**) BGS analysis of HIC1 promoter methylation in papillary thyroid cancer and matching adjacent noncancerous tissues (*n* = 40). Methylated sites are shown as filled circles, unmethylated sites as empty circles, and deletion or mutation sites as filled triangles. The percentage shown indicates the total methylation rate. The association between the level of HIC1 promoter methylation and HIC1 mRNA (**C**) or protein (**D**) expression in papillary thyroid cancer tissues was evaluated using a Pearson correlation analysis.

To assess the hypermethylation status of the *HIC1* promoter, a Bisulfite Genomic Sequencing (BGS) assay was performed. The positions of the BGS primers within the *HIC1* promoter are shown in Figure 2A. *HIC1* gene measurements in thyroid cancer and adjacent normal tissues were performed. *HIC1* cloning and sequencing confirmed the presence of more methylated CpG islands in the papillary thyroid carcinomas (61.3%) than the adjacent normal thyroid tissues (41.7%; *p <* 0.01, Figure 2B). Within the papillary carcinoma samples, there were also negative correlations between the amount of *HIC1* methylation and HIC1 mRNA expression (Figure 2C, *p <* 0.05), and between *HIC1* methylation and HIC1 protein expression (Figure 2D, *p <* 0.05). Consistent with the HIC1 immunohistochemistry data, the frequency of *HIC1* methylation in patients with lymph node metastasis was significantly higher than for patients without lymph node metastasis and for patients over 45 years-of-age (Table 2).

**Table 2:** Correlations between HIC1 promoter methylation and patient characteristics

Clinical feature	*n*	HIC1					
		Methylation frequency (%)	*P*	mRNA	*P*	protein	*P*
Lymph node metastasis							
Yes	18	68.1 ± 3.3	0.001	0.100 ± 0.001	0.001	0.191 ± 0.031	0.001
No	22	56.4 ± 2.8		0.322 ± 0.043		0.601 ± 0.037	
Sex							
Male	14	63.1 ± 2.6	0.089	0.233 ± 0.043	0.137	0.434 ± 0.072	0.320
Female	26	60.9 ± 4.6		0.217 ± 0.019		0.408 ±0.078	
Age (year)							
< 45	25	56.5 ± 3.7	0.001	0.302 ± 0.034	0.001	0.535 ± 0.097	0.001
≥ 45	15	70.4 ± 4.1		0.092 ± 0.019		0.221 ± 0.007	
Tumor size (cm)							
< 3	24	61.1 ± 2.4	0.147	0.241 ± 0.025	0.080	0.442 ± 0.043	0.061
≥ 3	16	62.5 ± 3.5		0.222 ± 0.042		0.480 ± 0.080	
TNM stage							
I–II	28	58.6 ± 2.1	0.001	0.274 ± 0.028	0.001	0.494 ± 0.051	0.001
III–IV	12	68.9 ± 1.8		0.101 ± 0.010		0.237 ± 0.023	
Capsule invasion							
Yes	17	62.9 ± 2.9	0.136	0.213 ± 0.019	0.193	0.387 ± 0.077	0.064
No	23	60.7 ± 5.3		0.231 ± 0.053		0.439 ± 0.089	

### HIC1 hypermethylation promoted SIRT1 expression in TPC-1 cells

**Figure 3:**
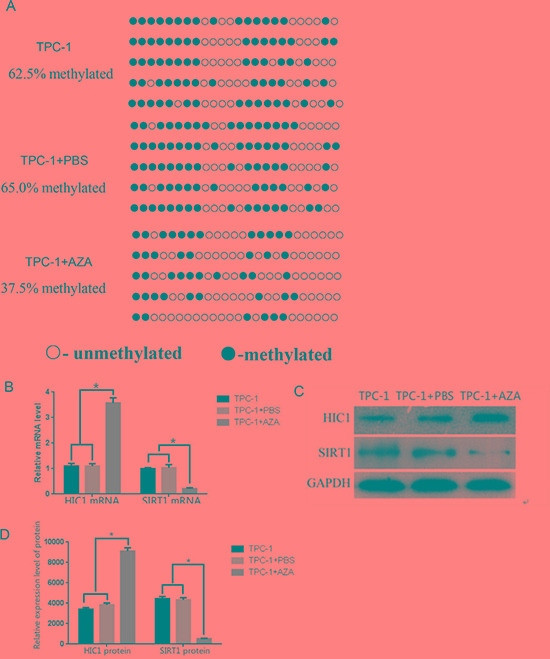
Effects of 5-aza-dC treatment on HIC1 and SIRT1 expression in TPC-1 cells (**A**) *HIC1* promoter methylation was assessed in the presence and absence of the demethylating agent 5-aza-dC using BGS analysis in a human papillary thyroid cancer cell line (TPC-1 cells). Methylated sites are shown as filled circles, unmethylated sites as empty circles, and deletion or mutation sites as filled triangles. The percentage shown indicates the total methylation rate. (**B**) The mRNA expression levels of HIC1 and SIRT1 were assessed by qRT-PCR in TPC-1 cells treated with 5-aza-dC. *p < 0.05 compared to untreated TPC-1 cells or TPC-1 cells + PBS using a Student's *t* test. (**C**) HIC1 and SIRT1 protein expression by Western blot (**D**) Quantification of HIC1 and SIRT1 protein expression in TPC-1 cells after 5-aza-dC administration (*n* = 3). *p < 0.05 compared untreated TPC-1 cells or TPC-1 cells + PBS using a Student's *t* test. GAPDH was used as an internal control.

Tissue analyses confirmed that the level of HIC1 expression in thyroid carcinoma tissue was greater than in normal tissue, and that within thyroid carcinomas there was a negative correlation between HIC1 and SIRT1 expression. Therefore, we hypothesized that silencing HIC1 would downregulate its expression, thereby upregulating SIRT1 expression. TPC-1 cells were treated with the demethylating agent 5-aza-dC and BGS amplification was used to measure methylation in the *HIC1* gene promoter. The TPC-1 cells treated with 5-aza-dC (TPC + 5-aza-dC, 37.5%) had significantly less methylation than untreated TPC-1 cells (TPC-1, 62.5%) and control TPC-1 cells treated with PBS (TPC-1 + PBS, 65.0%; Figure 3A, *p <* 0.05). RT-qPCR quantification confirmed that the TPC-1 + 5-azadC condition had the highest expression of HIC1 mRNA (Figure 3B, *p <* 0.05) and protein (Figure 3D–3E, *p <* 0.05), and the least SIRT1 mRNA and protein expression (Figure 3B and 3C, *p <* 0.05). These results demonstrated that reversing the *HIC1* promoter methylation upregulated *HIC1* expression and indirectly downregulated SIRT-1 expression, suggesting that in patients epigenetic changes in *HIC1* may affect HIC1/SIRT1 protein expression.

### Assessing the effects of 5-aza-dC treatment on cell cycle, apoptosis, cell senescence, and proliferation in TPC-1 cells

**Figure 4:**
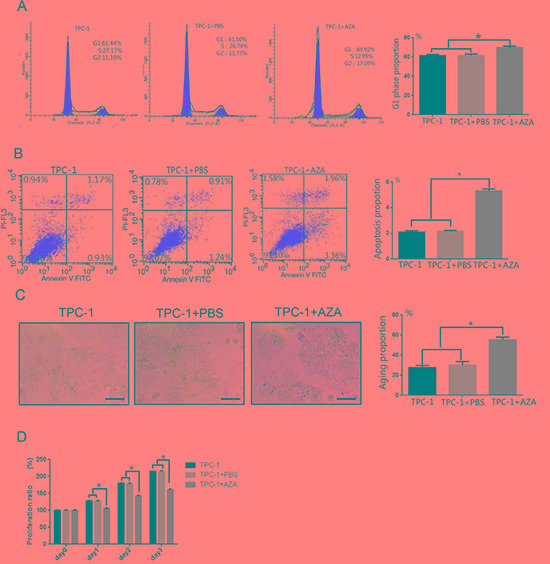
Effects of 5-aza-dC treatment on cell cycle, apoptosis, senescence, and proliferation in TPC-1 cells (**A**) Flow cytometry confirmed cell cycle arrest at the G1 phase after treatment with 5-aza-dC in TPC-1 cells. (**B**) AnnexinV staining indicated a significantly greater percentage of TPC-1 cells undergoing apoptosis after treatment with 5-aza-dC. (**C**) 5-aza-dC significantly increased the number of senescent (β-galactosidase positive) TPC-1 cells (100× magnification, Scale bar = 100 μm). (**D**) TPC-1 cell growth was inhibited after 5-aza-dC treatment. *p < 0.05 compared untreated TPC-1 cells or TPC-1 cells + PBS using a Student's *t* test.

A similar number of TPC-1 (61.44 ± 1.12%) and TPC-1 + PBS (61.50 ± 1.51%) cells were in the G1 phase of the cell cycle. Treatment with 5-aza-dC (69.92 ± 1.31%) increased the number of cells in the G1 phase (Figure 4A, *p <* 0.05) suggesting that 5-aza-dC induced demethylation of thyroid carcinoma cells arrested cells at the G1 phase. Annexin V-FITC/PI double staining confirmed a higher percentage of cells undergoing apoptosis in the TPC-1 + 5-aza-dC group (5.32 ± 0.15%) compared to the TPC-1 (2.10 ± 0.11%) and TPC-1 + PBS groups (2.15 ± 0.08%) (Figure 4B, *p <* 0.05). The lowest levels of senescence, measured as β-galactosidase staining, were detected in the TPC-1 group (27.52 ± 2.23%). The TPC-1 + PBS group (30.22 ± 3.42%) had a slightly greater percentage of senescent cells than the TPC-1 group. The TPC-1 + 5-aza-dC group (55.21 ± 2.72%) had the most senescent cells, indicating that demethylation increased cellular aging in papillary thyroid carcinoma cells (Figure 4C, *p <* 0.05). Cell proliferation was measured daily and the TPC-1 + 5-aza-dC group proliferated less than the TPC-1 and TPC-1 + PBS groups (Figure 4D, *p <* 0.05).

### Effects of forced HIC1 overexpression on SIRT1 expression, proliferation, cell cycle arrest, apoptosis, and cell senescence in TPC-1 Cells

**Figure 5:**
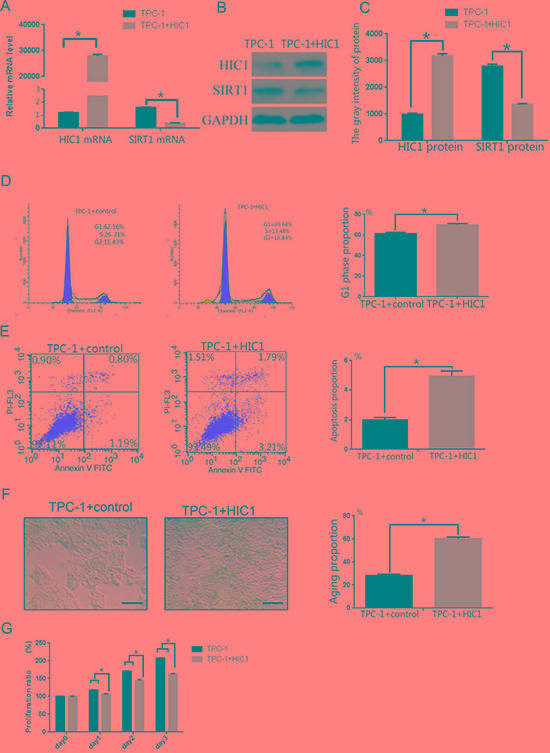
Effects of overexpression of pcDNA3.1(+)-flag-HIC1 on TPC-1 cells (**A**) qRT-PCR analysis of HIC1 and SIRT1 mRNA expression levels in TPC-1 cells after transfection with an HIC1 overexpression plasmid. (**B**–**C**) HIC1 and SIRT protein expression levels were determined in TPC-1 cells by Western blot (*n* = 3). GAPDH was used as an internal control. Flow cytometry was used to confirm that TPC-1 cells transfected with pcDNA3.1(+)-flag-HIC1 were arrested in the G1 phase (**D**) and that apoptosis was induced (**E**). (**F**) β-galactosidase staining showed that transfection with pcDNA3.1(+)-flag-HIC1 induced cellular senescence. Scale bar = 100 μm. (**G**) Growth of TPC-1 transfected with pcDNA3.1(+)-flag-HIC1 was inhibited. *p < 0.05 compared to the TPC-1 group transfected with control plasmid using a Student's *t* test.

We then measured expression of HIC1 and SIRT1 at the mRNA and protein levels after transfecting TPC-1 cells with the pcDNA3-FlagHIC1 plasmid (*HIC1* transfected group). Transfection with the plasmid increased HIC1 mRNA and protein expression compared to control TPC-1 cells (Figure 5A–5C, *p <* 0.05). In addition, the SIRT1 mRNA and protein expression levels were lowers in the *HIC1* transfected group than in untransfected cells (Figure 5A–5C, *p <* 0.05), showing that increased expression of HIC1 downregulated expression of the *SIRT1* gene in TPC-1 cells.

Consistent with the results obtained using 5-aza-dC treatment, the number of cells in the G1 phase in the *HIC1* transfected group (69.86 ± 1.22%) was slightly higher than in untransfected TPC-1 cells (62.36 ± 1.45%) (Figure 5D, *p <* 0.05). The levels of apoptosis (5.00 ± 0.16% vs 1.99 ± 0.11%, Figure 5E, *p <* 0.05) and β-galactosidase staining (60.32 ± 1.51% vs 28.23 ± 1.22%, Figure 5F, *p <* 0.05) were also greater in the HIC1 overexpressing cells than the control TPC-1 cells. Cell proliferation in the *HIC1* transfected group was significantly lower than in controls (Figure 5G, *p <* 0.05).

## DISCUSSION

Previous studies have shown that hypermethylation of the HIC1 promoter and overexpression of HIC1 protein occurs in thyroid cancer cells [22]; but this is the first report that HIC1 promoter methylation in papillary thyroid carcinomas correlates with lymph node metastasis, tumor stage, and patient age. HIC1 demethylation and exogenous transfection of an HIC1 expression plasmid upregulated HIC1 expression, inhibited cell proliferation, induced cellular aging, and increased cell cycle arrest in a papillary thyroid cell line.

The *HIC1* gene is distal to the p53 tumor suppressor gene and encodes a transcriptional repressor [14, 23], whose target genes are associated with cell proliferation [24], tumor growth [25], angiogenesis [26], tumor invasion [24, 27] and the epithelial-mesenchymal transition [13]. The *HIC1* gene is widely expressed in normal tissues but its expression in primary tumors varies (e.g., prostate [5–7], breast [11] and head and neck squamous cell carcinoma [28]) and is silenced by hypermethylation. Consistent with our results, a study published by Zhao et al. demonstrated that hypermethylation of *HIC1* is an important factor for progression of pancreatic carcinoma [10]. The significantly higher levels of *HIC1* methylation in papillary thyroid carcinomas compared to normal tissues suggests that excessive methylation is occurring in papillary thyroid carcinomas. Consistent with increased methylation, the levels of HIC1 mRNA and protein expression in papillary thyroid carcinomas were significantly lower than in adjacent normal tissues. The negative correlation between *HIC1* methylation and the expression of HIC1 mRNA and protein suggested that hypermethylation of the *HIC1* promoter inhibited *HIC1* expression, and may be associated with the development of papillary thyroid carcinoma.

Factors affecting the prognosis of papillary thyroid carcinoma include sex, age, tumor size, capsular invasion of the primary tumor, and metastasis to peripheral organs. We confirmed that patients with lymph node metastases had a higher frequency of *HIC1* methylation than patients without metastases. In addition, *HIC1* methylation frequency was significantly greater for patients older than 45 years-of-age and for those with higher TNM staging HIC1 mRNA and protein expression in patients with lymph node metastasis were significantly lower for those without lymph node metastasis and for patients younger than 45 years-of-age and for patients with higher TNM staging. Thus, *HIC1* methylation frequency and HIC1 mRNA and protein expression in papillary thyroid carcinoma, is closely associated with prognosis.

SIRT1, which is a class III histone deacetylase, protects cellular longevity in periods of oxidative stress and DNA damage, and is under the direct transcriptional control of HIC1 [16, 17]. HIC1 directly binds to the SIRT1 promoter and represses its transcription; thus the loss of HIC1 leads to the accumulation of SIRT1 [16, 17]. Studies indicate that SIRT1 is overexpressed in various human malignancies such as breast [29] and prostate cancers [30]. Consistent with these studies, we found higher levels of SIRT1 mRNA and protein expression in papillary thyroid carcinomas than in the adjacent normal thyroid tissues. Notably, there was also a negative correlation between HIC1 and SIRT1 expression. In papillary thyroid carcinoma, it is likely that reduced expression of the *HIC1* gene contributes to upregulation of SIRT1 expression. We also found that forced demethylation of the *HIC1* promoter using 5-aza-dC or overexpression of HIC1 suppressed expression of SIRT1. Inhibition of SIRT expression was associated with inhibited proliferation and the induction of cellular aging and cell cycle arrest. These findings indicated that *HIC1* methylation regulated the HIC1/SIRT1 signaling pathway and was involved in the development of papillary thyroid carcinomas.

In summary, hypermethylation of the *HIC1* promoter in thyroid papillary carcinoma might contribute to aberrant expression of HIC1/SIRT1, which in turn promotes thyroid cancer. Hypermethylation of the *HIC1* promoter and aberrant expression of HIC/SIRT1 may be useful for assessing the risk of developing thyroid papillary carcinoma and may be a novel therapeutic target.

## MATERIALS AND METHODS

### Tissue specimens

All tissue specimens were surgically collected from the Department of General Surgery, the Second Affiliated Hospital, Fujian Medical University in Fujian, China from March 2013 to March 2014. This study was approved by the Ethics Committee of the Second Affiliated Hospital of Fujian Medical University, and was performed in accordance with the ethical standards laid down in the 1964 declaration of Helsinki and all subsequent revisions. All of the patients enrolled in the study provided informed consent prior to their inclusion in the study. Papillary thyroid carcinomas (*N* = 40) and matching normal (confirmed histologically by a pathologist) thyroid tissues from the opposite lobe were collected from the same patient. TNM staging of the thyroid tumors was performed according to the 2012 Guidelines of the Union for International Cancer Control (UICC) and the American Joint Committee on Cancer (AJCC). Post-operative tumor specimens were histopathologically confirmed as papillary thyroid carcinomas. All patients had normal preoperative thyroid function and complete clinical and pathological data. None of the patients received preoperative chemotherapy or radiotherapy. Tumor specimens were divided into three equal portions for histopathological examination, Western blot analysis after preservation at −80°C, and real-time fluorescent-based quantitative PCR (RT-qPCR) and quantification of methylated CpG islands after preservation in RNAlater at 4°C.

### RNA isolation and RT-PCR analysis

TRIzol (Invitrogen, Carlsbad, CA) was used to isolate total RNA from cultured cells or flash-frozen thyroid tissues. The RevertAid H Minus First Strand cDNA Synthesis Kit (MBI, Canada) with random primers was used to synthesize the cDNA. PCR reactions involved 0.5 μL of cDNA and the ABI PRISM 7500 Sequence Detection System (Applied Biosystems, Foster City, CA, USA).

*Homo sapiens* 18 S ribosomal RNA (18 s rRNA) gene was used as internal reference gene (GenBank Accession No.: NR_003286). The primer sequences utilized were: 18 s rRNA forward primer: CCTGGATACCGCAGCTAGGA and 18s rRNA reverse primer: GCGGCGCA- ATACGAATGCCCC; HIC1 forward primer: CCCGGGACTGATAATGTGA and HIC1 reverse primer: AGACCTGGTGGTAGGCTCTT; and SIRT1 forward primer: ACTTCAGGT- CAAGGGATGG and SIRT1 reverse primer: GTTCTGGGTATAGTTGCGAAG. Relative quantification of each gene was calculated using the formula: C_T_ = (C_T.Target_ − C_T.18srRNA_)_Time x_ − (C_T.Target_ − C_T.18s rRNA_)_Time 0_. RQ value = 2^-CT^. T.target represents target gene (HIC1 or SIRT1); T.18s rRNA represents internal reference gene (18 s rRNA); Time *x* represents pathological specimen; and Time 0 represents tumor-adjacent normal tissue. The RQ value is the relative expression level of the target gene to the reference gene.

### Immunohistochemistry

To evaluate HIC1 and SIT1 protein expression, immunohistochemistry was performed on 4 μΜ-thick tissue sections from the thyroid papillary carcinomas and paired adjacent normal tissues as previously described [31]. Sections were incubated with a primary antibody against HIC1 (1:100) or SIRT1 (1:150) at 4ºC overnight and incubated with horseradish peroxidase-conjugated goat anti-mouse/rabbit IgG antibody (ZSGB-Bio, Beijing, China) followed by 3, 3′-diaminobenzidine tetra-hydrochloride (DAB) staining. To quantify the level of nuclear HIC1 or SIRT1 staining, the percent of stained cells were scored as: 0 = no staining; 1 = 1–25% of nuclei stained; 2 = 25–50% of nuclei stained; and 3 = > 50% of nuclei stained. Samples scoring 0–1 were considered negative and samples staining 2–3 were considered positive.

### DNA extraction and bisulfite genomic sequencing

Tissue or cellular genomic DNA was extracted using the Promega wizard genomic DNA purification kit, and then used as a template for the BGS assay using the QIAGEN EpiTect Bisulfite Kit following the manufacturer's protocol. The sequences of the *HIC1* upstream and downstream PCR primers were designed using MethPrimer (http://www.urogene.org/methprimer/) and were: 5′-CGTTAGGGTTGCGGGAA-3′ and 5′-GCCCTCCCACCTATACCCACCTAAAA-3′ respectively. The BSP-HIC1 sequence fragment size was 386 bp (including 24 CG sites). The PCR reaction conditions were: a denaturing step at 95°C for 5 min, followed by 40 cycles of 95°C for 15 s and 60°C for 15 s, and finally extension at 72°C for 8 min. PCR products were separated using agarose gel electrophoresis, purified with a QIAquick gel extraction kit (Qiagen, Valencia, CA, USA) and then cloned into a vector for sequencing. The methylation status of HIC1 was quantified by counting the number of methylated CpG sites in all the clones and the percentage of methylated CpG sites was determined from all of the CpG sites.

### 5-aza-dC treatment

The TPC-1 cell line was obtained from the Institute of Interdisciplinary Research (IRIBHM) (Brussels, Belgium) and maintained in RPMI-1640 medium (Gibco, Paisley, Scotland, UK) supplemented with 10% bovine calf serum (Gibco). As described in previously [10], the cells were incubated in culture medium with 5-aza-dC (1 μg/mL) for 4 days to assess cell senescence, cell cycle and apoptosis, and 5 days to measure cell proliferation. The culture medium was changed on days 1 and 3. PBS was used as control. Cells were collected at the end of the fourth day for genomic DNA and protein extraction.

### Transfection

pCDNA3.1(+)-flag or pcDNA3.1(+)-flag-HIC1 (1 μg) that contained full-length human HIC1 was transfected into TPC-1 cells grown in 24-well tissue culture plates using Lipofectamine 2000 (Invitrogen, Carlsbad, CA). After 36 h, the cells were collected and the mRNA and protein expression levels of HIC1 or SIRT1 were measured as was as cell proliferation, cell cycle arrest, and senescence.

### Cell proliferation, cell cycle, and apoptosis analyses

TPC-1 cells treated with 5-aza-dC or transfected with an HIC1 expression plasmid were analyzed using a cell cycle detection kit or Annexin V-FITC kit (Beckman Coulter Inc, Fullerton, CA) as previously described [32]. For the proliferation assay, cells were incubated in a CCK8 solution (Beyotime, China) for 0, 24, 48, or 72 h and the OD_450_ was recorded according to standard methods.

### Senescence-associated β-galactosidase (SA-β-gal) assay

SA-β-gal activity was measured using a β-Galactosidase staining kit (Beyotime, China) as described previously [33]. TPC-1 cells were washed and fixed for 15 min and incubated overnight at 37°C in the staining solution. Green-stained images were analyzed by confocal laser scanning microscopy.

### Statistical analysis

Statistical analysis was performed using SPSS version 16.0 (SPSS Inc., Chicago, IL). Data are presented as the means ± SEM, unless otherwise indicated. A two-sided *p value* *<* 0.05 was considered statistically significant. Each experiment was performed at least three times.
